# Sphere Drag and Heat Transfer

**DOI:** 10.1038/srep12304

**Published:** 2015-07-20

**Authors:** Zhipeng Duan, Boshu He, Yuanyuan Duan

**Affiliations:** 1 Institute of Combustion and Thermal System, School of Mechanical, Electronic and Control Engineering, Beijing Jiaotong University, Beijing 100044, China; 2 Key Laboratory of Thermal Science and Power Engineering of MOE, Beijing Key Laboratory for CO2 Utilization and Reduction Technology, Tsinghua University, Beijing 100084, China

## Abstract

Modelling fluid flows past a body is a general problem in science and engineering. Historical sphere drag and heat transfer data are critically examined. The appropriate drag coefficient is proposed to replace the inertia type definition proposed by Newton. It is found that the appropriate drag coefficient is a desirable dimensionless parameter to describe fluid flow physical behavior so that fluid flow problems can be solved in the simple and intuitive manner. The appropriate drag coefficient is presented graphically, and appears more general and reasonable to reflect the fluid flow physical behavior than the traditional century old drag coefficient diagram. Here we present drag and heat transfer experimental results which indicate that there exists a relationship in nature between the sphere drag and heat transfer. The role played by the heat flux has similar nature as the drag. The appropriate drag coefficient can be related to the Nusselt number. This finding opens new possibilities in predicting heat transfer characteristics by drag data. As heat transfer for flow over a body is inherently complex, the proposed simple means may provide an insight into the mechanism of heat transfer for flow past a body.

The behavior of systems involving the motion of small particles relative to fluids in which they are immersed covers a wide range of phenomena of interest to both scientists and engineers. The fluid dynamic drag on a sphere and the terminal settling velocity of a single spherical particle in an infinite fluid are of interest in numerous fields. As such, they have been the subjects of many experimental and theoretical investigations. One might suppose that all the fundamental problems had been solved long ago. It has important physical and mathematical meanings to have an analytic drag formula. However, this famous classical problem seems quite difficult. Because the flow is highly complicated, the drag coefficient cannot be expressed in an analytical form for a wide range of Reynolds numbers. Since the milestone works of Stokes, Oseen, and others for flow past a sphere, investigations on the drag of spheres have been made over a very wide range of Reynolds numbers and numerous sphere drag correlations have been proposed based on experimental data^1^,^2^,^3^,^4^,^5^,^6^,^7^,^8^,^9^,^10^,^11^,^12^,^13^; to mention but some. The objective of this paper is to furnish the scientists and engineers with a simple and direct means of estimating drag past a sphere and the original physical behavior can be clearly reflected. Furthermore, it is disclosed that a relationship in nature between the sphere drag and the convection heat transfer may exist.

## Results

When a body is moved with a uniform velocity along a straight line through a fluid at rest, it experiences a force in a direction opposite to that of the motion. This force is called the drag or resistance. Historically, the first resistance law was proposed by Newton, the founder of mechanics. This law still holds today for motions where the drag is due to inertia. One of the most fundamental problems in fluid dynamics is that of drag of bodies moving through fluids. It is common, due to historical reasons, to define a dimensionless drag coefficient *C*_*D*_ as follows:


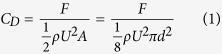


where *F* is the drag force on the particle; *U* is the relative velocity of the particle with respected to the fluid; ρ is fluid density; *d* is the diameter of particle; *A* is the projected area of the body in the direction of the flow. Dimensional analysis shows that the drag coefficient is a function of the Reynolds number (Re). The drag at very low Reynolds number was predicted by Stokes on the assumption that the inertia terms in the equation of motion can be neglected. An analytical solution for the drag coefficient at the creeping flow limit is known as Stokes solution:


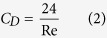


**Figure 1:**
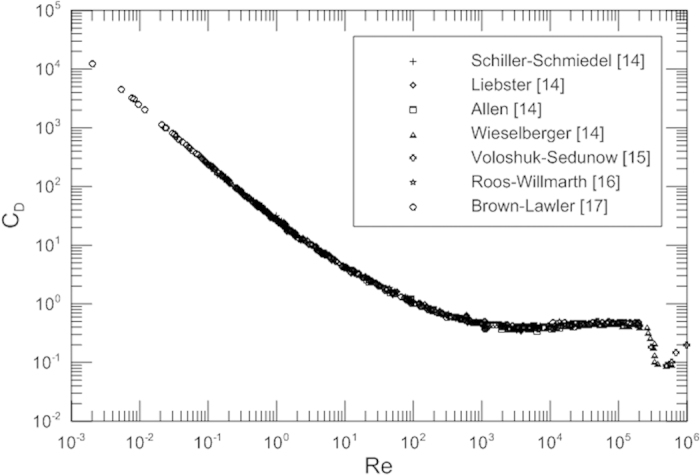
Customary drag coefficient for spheres as a function of the Reynolds number.

It is noted that this introduces a Reynolds number effect into a regime that is in fact independent of Reynolds number. It is common to nondimensionalize *F* by introducing the dynamic pressure *ρU*^2^/2, which is a specious idea for creeping flow. Attempts have been made to deduce a more general relation for the drag of a sphere at small Reynolds numbers than that obtained by Stokes, by taking some account of the inertia terms in the equations of motion. The idea of using creeping flow to expand into the large Reynolds number region is not successful. The drag coefficient and Reynolds number relation can be ascertained only by experiment. Figure 1 represents the customary drag coefficient of spheres as a function of the Reynolds number. The extensive historical experimental points collected by Schlichting^14^ and other experimental data from Voloshuk and Sedunow^15^, Roos and Willmarth^16^, Brown and Lawler^17^ for the drag coefficient of spheres of widely differing diameters fall on a single curve. This figure displays the classic shape of the drag curve for spheres and appears in every fluid mechanics and heat transfer text. But this does not mean that we know and understand drag coefficient well. It is expected that next discussion shed some light on the physical meaning of appropriate drag coefficient. It is noted that the customary drag coefficient decreases with an increase of the Reynolds number. The drag and the customary drag coefficient have an opposite trend. With increasing Reynolds numbers the drag coefficient first decreases, but with a still further increase of the Reynolds number the value of drag coefficient slightly increases again. It is also noticed that there is a minimum for the drag coefficient in a Reynolds number value around 5000 before the boundary layer transition from laminar to turbulent. Then the drag coefficient slightly increases and remains constant (independent of Reynolds numbers) with an increase of the Reynolds number up to 2.0 × 10^5^; however, it is well known that this is a regime where *C*_*D*_ is dependent on the Reynolds number. This strange variation trend of drag coefficient with Reynolds number is a further proof that the customary drag coefficient possibly is not an appropriate dimensionless parameter to represent the drag.

The proper drag coefficient should scale as ~*F*/*μUd*, where *μ* is the dynamic viscosity, but nearly everyone uses the inertia type of definition *C*_*D*_ = 2*F*/*ρU*^2^*A* which was first derived and proposed by Newton. We therefore propose a different dimensionless parameter which is more closely associated with the drag. The drag is related to the appropriate drag coefficient in the following manner:


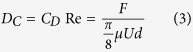


The above grouping *D*_*C*_ is interpreted as the dimensionless drag. It will be shown that the appropriate drag coefficient has more physical meaning and significance than the customary drag coefficient.

Equation (3) is more appropriate and convenient to calculate drag especially for Stokes flow problems as *D*_*C*_ is a constant. It is not necessary to obtain the value of customary drag coefficient *C*_*D*_ although it is traditional to use customary drag coefficient to calculate drag due to historical reasons.

**Figure 2:**
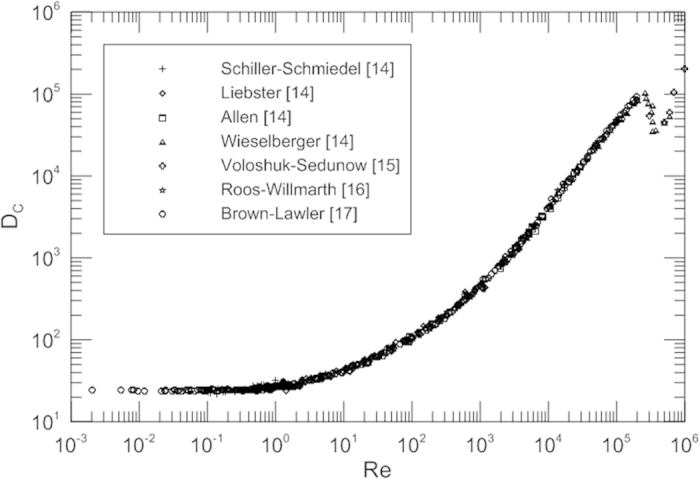
Appropriate drag coefficient for spheres as a function of the Reynolds number.

Figure 2 represents the appropriate drag coefficient of spheres as a function of the Reynolds number. It is noted that the appropriate drag coefficient increases with an increase of the Reynolds number. It is observed that the drag and the appropriate drag coefficient have a same trend. Figure 2 serves as a great visual aid in the drag analysis. The curve is very smooth and there exist no a minimum for the appropriate drag coefficient before the boundary layer transition from laminar to turbulent.

It is obvious that the customary drag coefficient *C*_*D*_ is not a desirable dimensionless parameter to describe fluid flow behavior as the variation trend with Reynolds number is peculiar and *C*_*D*_ is unnecessary and superfluous especially when dealing with Stokes flow problems. Furthermore, it is noted that the customary drag coefficient *C*_*D*_ decreases with an increase of Reynolds numbers and approaches a constant or zero as *Re*→∞; however, the dimensionless drag (*D*_*C*_) increases with an increase in the Reynolds number and is a desirable dimensionless parameter to reflect a natural tendency and truly represent drag. It is seen that the appropriate drag coefficient curve is smoother than the customary drag coefficient curve. Figure 2 is proposed to calculate the dimensionless drag coefficient and drag. This figure is more reasonable and intuitive to reflect the original physical behavior; a change in the Reynolds number causes a change in the appropriate drag coefficient as well as the drag.

Heat transfer at the surface of spheres is determined by the flow conditions. A laminar boundary layer covers the upstream portion of the surface, and this boundary layer separates from the side of the sphere, creating an irregularly fluctuating flow condition along the downstream portion. On the side of the sphere the boundary layer may become turbulent at large Reynolds numbers. For the average heat transfer coefficient between a spherical surface and a free stream, Whitaker^18^ proposed the Nusselt number correlation


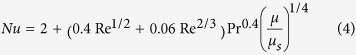


where *Pr* is the Prandtl number and *μ*_*s*_ is the dynamic viscosity evaluated at the surface temperature. This correlation is valid for 0.71 ≤Pr ≤ 380, 3.5 ≤Re ≤ 7.6×10^4^ and 1.0 ≤(*μ*/*μ*_*s*_) ≤ 3.2. When compared with experimental data, this formula seems to agree with both liquid- and gas-flow data. Numerous heat transfer correlations have been proposed. Ranz and Marshall^19^ provide a simple correlation for the Nusselt number based on experimental data. The correlation of Ranz and Marshall^19^ and the relation proposed by Whitaker^18^ provide comparable accuracies in similar ranges of the Reynolds number. The correlation of Ranz and Marshall^19^ is, therefore, also recommended due to its simplicity. It is worth noting that the creeping flow limit of the formula, *Nu* = 2, is heat transfer by conduction from a spherical surface to a stationary, infinite medium around the surface.

There are certain common principles and laws that apply to the momentum and energy transport processes. It is speculated that the analogies and similarities exist between the dimensionless drag (the appropriate drag coefficient) and the dimensionless mean wall heat flux. It is expected that one transport phenomenon can be related to another transport phenomenon. It would also be very insightful to express the heat transfer in terms of drag.

The analogy between drag and heat transfer rigorously holds for the Stokes flow and may approximately hold for the high Reynolds number flow, which is useful in obtaining a first approximation for the heat transfer


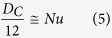


The above correlation offers simplicity in computation, and the price we pay for this convenience is some loss in accuracy. The accuracy of equation (5) depends on having Prandtl number approximately equal to unity and the temperature influence on properties is not significant (*μ*/*μ*_*s*_ ≈1). Equation (5) is satisfactory for gases in which *Pr* is approximately unity. Furthermore, it is shown that the analogy can be applied over a wide range of *Pr* if appropriate modifications are added in accordance with experimental data


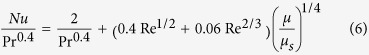


The Nusselt number may be obtained from the analogy


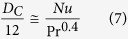


**Figure 3:**
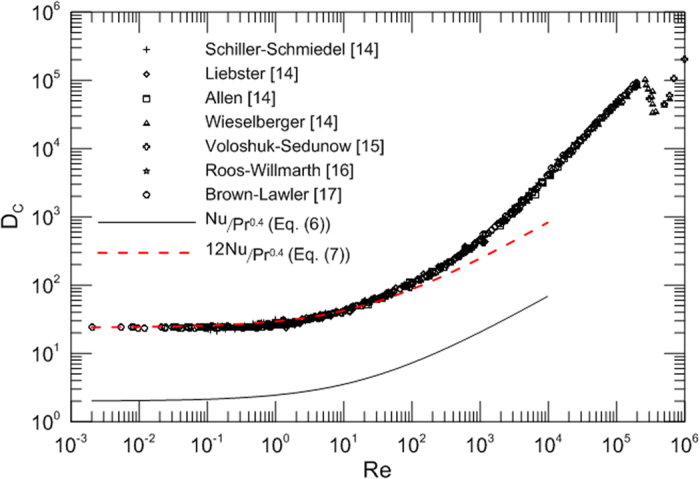
Comparison of drag experimental data and heat transfer correlation.

The above relationship offers reasonably accurate and simplicity in predicting heat transfer. The above model for smooth sphere is presented as *Nu*/Pr^0.4^ and it is compared with the extensive hydrodynamic drag data collected by Schlichting^14^ and other researchers Voloshuk and Sedunow^15^, Roos and Willmarth^16^, Brown and Lawler^17^ as shown in Fig. 3. The model is seen to be strictly qualitative. It is interesting to observe that the Nusselt number and the appropriate drag coefficient increase with an increase of the Reynolds number and have a similar trend and the speculation is surprisingly successful.

It is possible to derive correlations for heat and mass transfer from hydrodynamic drag experiments. The dimensionless drag may be related to the dimensionless mean wall heat flux (Nusselt number), which provides a good insight into the transport phenomena mechanism of flow past a body. This could be significant because of a shortage of information for the Nusselt number for most non-spherical bodies in the literature. The role played by the heat flux has similar nature as the drag. The proposed model does provide a means to predict the Nusselt number for flow over other body shapes.

**Figure 4:**
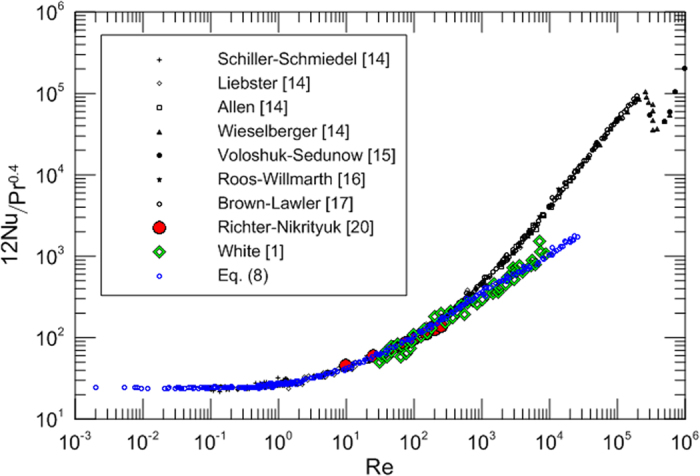
Comparison of experimental data for drag and heat transfer.

Richter and Nikrityuk^20^ numerically investigated heat transfer for flow past spherical particles at Reynolds numbers between 10 and 250. Using the historical drag experimental data we obtain the results as plotted on Fig. 4 in comparison with heat transfer results for spheres to elucidate the relationship between drag and heat transfer. The heat transfer numerical data from Richter and Nikrityuk^20^ and the historical heat transfer experimental data collected by White^1^ are included in this figure. An interesting feature is shown in Fig. 4 and good agreement is observed when the model is compared with published values for Nusselt numbers. It is seen that the model is surprisingly successful and the deviation is very small and negligible for relatively small Reynolds numbers. This could be significant because many engineering applications reside squarely within the low end of the Reynolds number scale. The use of the model provides remarkable representation of the data from Richter and Nikrityuk^20^. Some of the deviation from the collected experimental results^1^ for large Reynolds numbers could be due to experimental error; the accuracy of equation (7) can be improved by accounting for the Reynolds number effect:


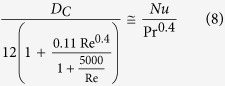


Figure 4 presents the comparison between the proposed models of equations (7 and 8) and the extensive drag and heat transfer experimental data of flow past a sphere and may be used as a first approximation of the Nusselt number. Based on the boundary layer analogy between temperature boundary layer and concentration boundary layer, we can deduce the correlation to the mass convection problem by substituting Sherwood number in place of Nusselt number and Schmidt number in place of Prandtl number.

The sphere is the simple geometry of body immersed in a uniform flow. As experimental works about the Nusselt number relations for non-spherical particles are rare and no models or graphical and tabulated data exist for most geometries of bodies, the proposed means can be used to predict heat transfer based on drag experimental data for flow past other bodies of shape. The characteristics of the flow past circular cylinder placed transversely in a fluid stream, in general, is different from that of sphere. However, it is found that there is a great similarity in the development of dimensionless drag and Nusselt number between the cylinder and the sphere. We would expect the analysis to be accurate for bodies of nearly spherical shape. The estimate can be possibly used in analyses of many three-dimensional body shapes.

It is of some interest to examine the possibility that fluid may slip at the surface of the sphere. There are cases of practical interest, where interface slip has been observed such as rarefied gas flows. This problem was first solved by Basset^21^. The most plausible hypothesis one can frame under these circumstances is that the tangential velocity of fluid relative to the solid at a point on its surface is proportional to the tangential stress prevailing at that point. The subject of heat and mass transfer from very small spheres has attracted considerable attention in the recent years with the applications of nanofluids where the velocity slip at the fluid-solid interface is expected. Following previous work by Basset^21^, the appropriate drag coefficient in the creeping flow limit is


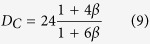


where β is the slip coefficient. In the case of perfect slip, *β*→∞, the drag is less than in Stokes theory by a factor of 2/3.

There are very few experimental data on slip flow heat transfer past a microsphere because it is quite difficult to measure local temperature and control thermal boundary conditions. It is still a challenge to experimentally investigate slip flow heat transfer past a microsphere for the research community. A survey of the available literature indicates a shortage of information for slip flow heat transfer past a microsphere. It is possible in a way to derive correlations for heat and mass transfer from hydrodynamic drag experiments. The dimensionless drag may be related to the dimensionless mean wall heat flux, which provides a good insight into the mechanism of slip flow heat transfer past a sphere. The proposed model does provide a means to predict the Nusselt number for slip flow over a sphere in the absence of experimental data.

## Discussion

Historical drag and heat transfer experimental data for flow past a sphere are critically examined. The appropriate drag coefficient is proposed to predict the drag for flow past a sphere. It is found that the dimensionless drag (*D*_*C*_) is a more desirable dimensionless parameter to intuitively describe fluid flow behavior so that fluid flow problems can be solved in a simple and direct manner. The dimensionless drag is presented graphically, and it is more general and reasonable to represent the original fluid flow physical behavior than the traditional century old drag coefficient Reynolds number relation curve. It is observed that a relationship in nature between the sphere drag and the convection heat transfer may exist, which provides a good insight into the underlying physical mechanism. The drag data can be utilized to obtain an initial engineering approximation of the Nusselt number and successful analogy and approximation is an art. Simple model is proposed for predicting heat transfer for flow past a sphere. The present model takes advantage of the selection of a more appropriate dimensionless drag to develop simple models. Given that some of the deviation is probably due to experimental error rather than to failure of the relations to represent the physical behavior, the proposed means are probably reliable for most non-spherical bodies of shape. It is therefore recommended as more convenient and accurate for obtaining a first approximation of the heat and mass transfer for flow past a body.

There are not any experimental data in the literature with surface slip over a sphere. Because slip flow heat transfer past a sphere is inherently complex, this proposed means may provide much of the information and insight about the nature of slip flow heat transfer past a sphere. It is noted that although there has been little work in slip flow heat transfer, in the non-continuum regime, there has been considerable work on the drag and mass transfer rates to aerosol nanoparticles. Zhang *et al*.^22^ and Gopalakrishnan *et al*.^23^ similarly utilized non-traditional dimensional analysis approaches to find almost universal relationships for the mass transfer rate to a small nonspherical particle and for the Knudsen number dependent drag force on a particle. Zhang *et al*.^22^ and Gopalakrishnan *et al*.^23^ used a combined approach to systematically examine low Reynolds number drag forces on nonspherical aerosol particles in the momentum transfer transition regime. Two approximations are first invoked to define appropriate size descriptors for nonspherical particles in the continuum and free molecular limits. They used dimensional analysis to reveal a suitable functional form for both the Knudsen number and the dimensionless friction factor, which describes orientationally averaged drag across the entire Knudsen number range. Zhang *et al*.^22^ and Gopalakrishnan *et al*.^23^ justifies that mass transfer and momentum transfer of vapor and gas molecules to particles of arbitrary shape are linked; measurement of drag forces on particles enables prediction of the condensation rates onto particles, and vice versa. This is a further proof of the reliability of the present study.

In addition, a general analogy between heat and mass transfer is likely to be common everywhere. If this is so, mass transfer can be estimated if heat transfer can be. The proposed models are useful because they allow information gained with a theoretically and experimentally relatively “simple” physical phenomenon to be applied to the analogous physical phenomena whose theoretical and experimental treatment is extremely difficult. A simple method is desired that, in addition to overcoming mathematical and experimental difficulties, gives relatively accurate answers easily for quite complex situations, even though the answers are not exact. It is appropriate not to draw rigorous conclusions on the analogy and the accuracy of the proposed models because there is plenty of room for improvement. The analogy between drag and heat transfer should hold and drag and heat transfer should show the same tendency. This paper cultivates the facility to perform the kind of analysis which, although not exact, still provides useful information concerning real engineering needs. This paper provides a sound basis for studying the relationship between drag and heat and mass transfer. With some relatively simple ideas from knowledge, observation, and intuition, one can predict some fairly complex transport phenomena to make the complex simple without losing the essence.

In conclusion, it has been demonstrated that the customary drag coefficient is not the best choice to represent the original fluid flow physical behavior. We find a remarkable relationship in nature between the sphere drag and heat transfer. It has also been shown that the use of the appropriate drag coefficient yields excellent similarity between drag and heat transfer for flow past a sphere. The appropriate drag coefficient is the more suitable dimensionless drag than the customary drag coefficient proposed by Newton. The commonly used century old customary drag coefficient should be retired and replaced by the appropriate drag coefficient.

## Additional Information

**How to cite this article**: Duan, Z. *et al*. Sphere Drag and Heat Transfer. *Sci. Rep*. **5**, 12304; doi: 10.1038/srep12304 (2015).
